# Supplementary material for: Dynamics of replication origin over-activation
Source: Nat Commun. 2021 Jun 8;12:3448. doi: 10.1038/s41467-021-23835-0 (PMC8187443; doi:10.1038/s41467-021-23835-0)
Supplement: Supplementary file 1 — Supplementary Information [file 41467_2021_23835_MOESM1_ESM.pdf]

Supplementary Information

DYNAMICS OF REPLICATION ORIGIN OVER-ACTIVATION

Fu et al.

Supplementary figures

Supplementary tables

Supplementary methods

Supplementary Figure

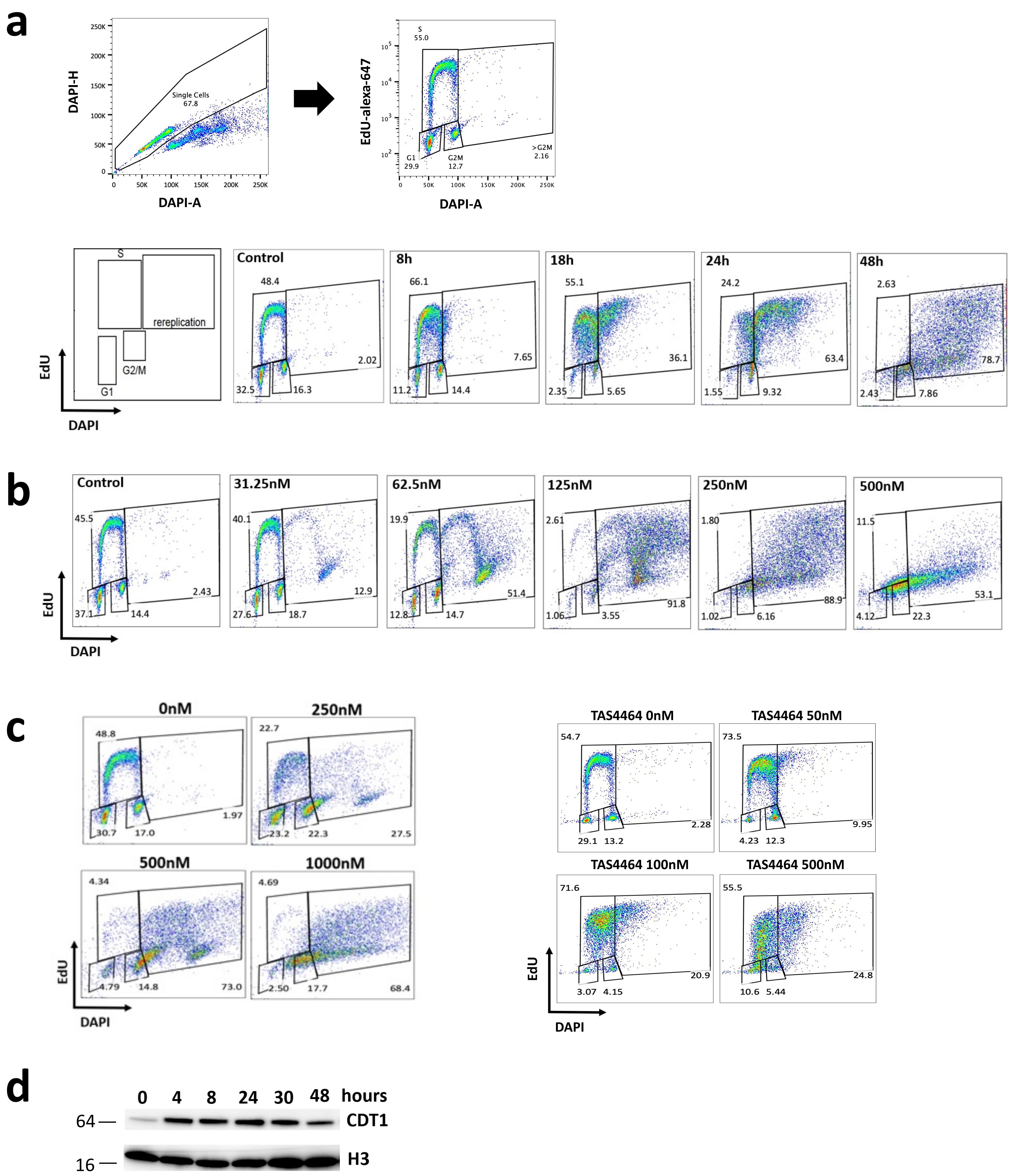

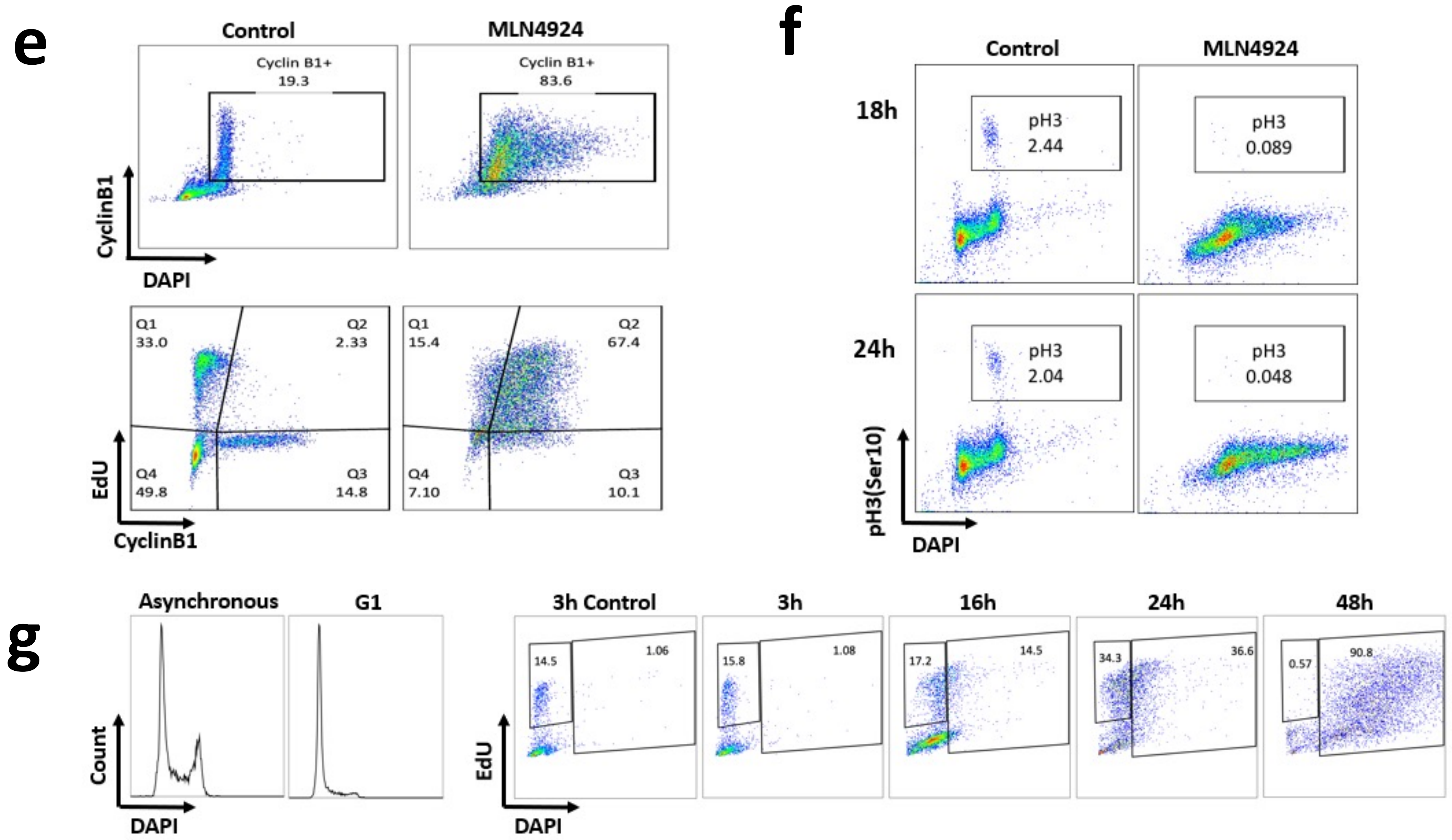

Supplementary Fig. 1 MLN4924 induces massive re-replication accompanied with increased CDT1. a Top, single cells gated by DAPI-A and DAPI-H as shown were used for all the flow cytometry analysis (an example of 2 dimensional cell cycle graph shown on the right) shown in this study. Bottom left, schematic representation of cell distribution at the different cell cycle stages (EdU indicating DNA synthesis, DAPI indicating DNA content). Re-replicating cells are cells with DNA content higher than G2/M cells that exhibit EdU incorporation. Bottom right, cell cycle profiles of HCT116 cells treated with 250nM MLN4924 for different time periods. **b** Cell cycle profiles of HCT116 cells treated with the indicated doses of MLN4924 for 48h. **c** Cell cycle profiles of U2OS cells treated with the indicated concentration of MLN4924 for 48h (left) or TAS4464 for 24h (right). **d** HCT116 cells were treated with 250nM of MLN4924 for the indicated times and CDT1 levels were detected by immunoblotting. 2 biological repeats show similar results. **e** Flow cytometry to monitor cell cycle progression and cyclinB1 levels. CyclinB1 positive cells were significantly increased and kept replicating (EdU positive) in re-replicating cells treated with 250nM of MLN4924 for 24h. **f** Flow cytometry detected pH3(Ser10) positive (mitotic) cells. Almost no pH3(Ser10) positive cells detected in MLN4924 treated HCT116 cells for 18h and 24h. **g**. HCT116 cells were elutriated by a centrifugal elutriator to isolate G1 cells, then G1 cells were treated with 250nM of MLN4924 for the indicated time. Cell cycle were monitored by flow cytometry. Asynchronous, G1, and 3 hour control (3h C) cells are used as controls.

a

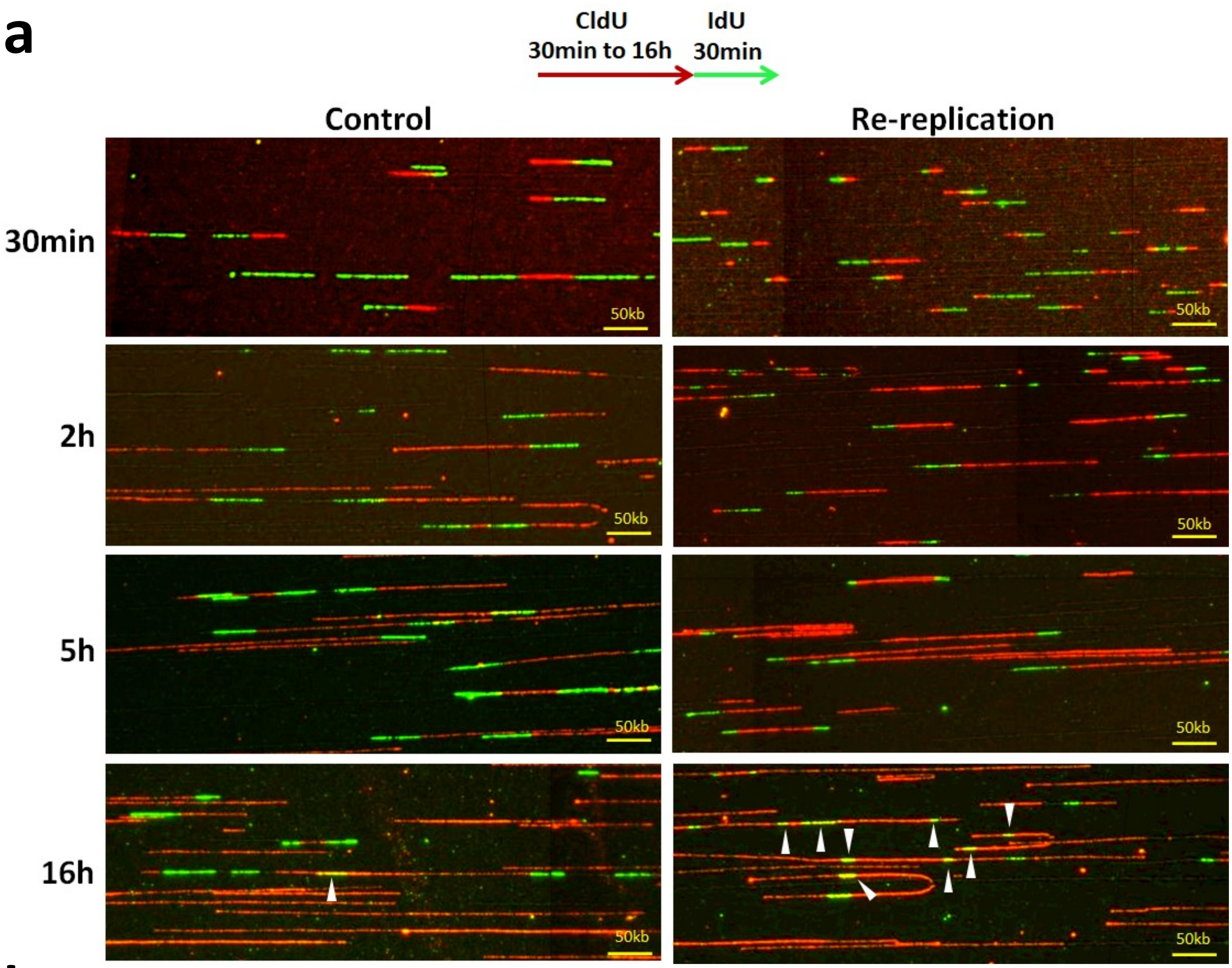

b

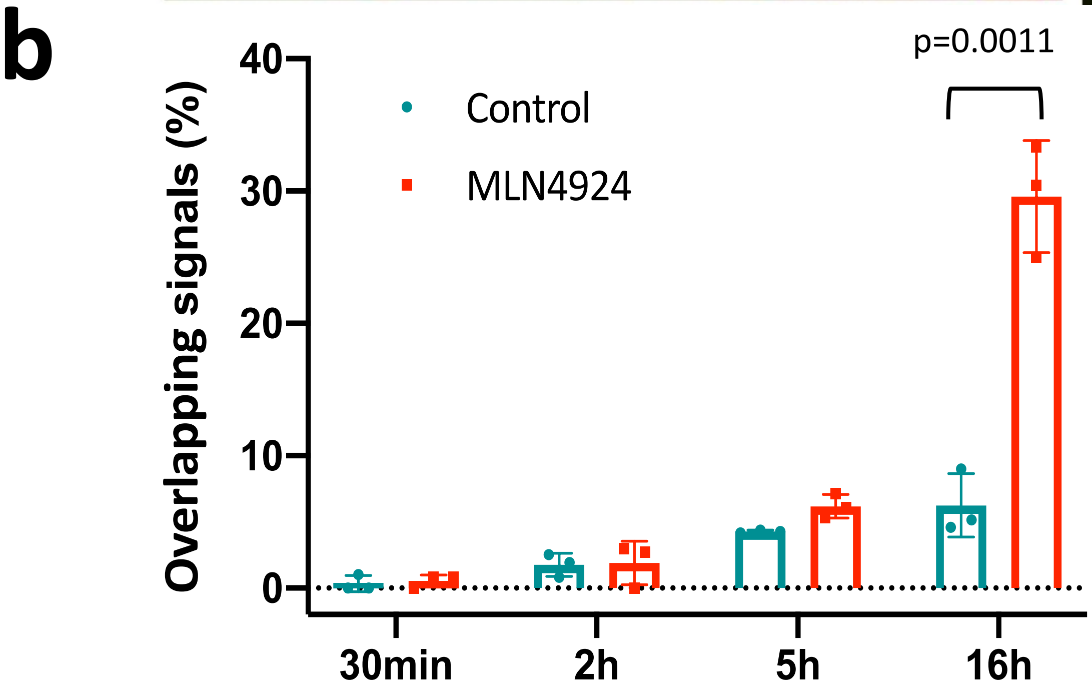

	30min	2h	5h	16h
Control	0.0 (90)	2.5 (118)	4.2 (96)	5.2 (116)
	1.0 (96)	1.9 (103)	4.3 (140)	9.0 (89)
	0.0 (91)	0.8 (122)	4.4 (91)	4.6 (109)
MLN4924	0.8 (124)	2.7 (111)	7.1 (112)	30.4 (115)
	0.8 (123)	3.0 (100)	5.3 (131)	33.3 (114)
	0.0 (140)	0.0 (100)	6.1 (132)	25.0 (88)

Supplementary Fig. 2, Colocalization of re-replication signals after prolonged labeling. HCT116 cells were treated with 250nM of MLN4924 for 24h. To test when re-replication signals coincide with replication signals obtained during the first round of replication, CldU was added to cells for the indicated times, then IdU for 30min before fibers were collected and processed by DNA combing. **a** Representative images are shown, white triangles indicate yellow tracks, indicating the colocalization of signals obtained during the first round of replication and re-replication. **b** Frequency of CldU and IdU overlapping (yellow) tracks. The number of overlapping tracks were counted in images from 3 randomly selected. Percentages of yellow signals (mean with SD) from each sample are shown. p=0.0011 by an unpaired two-sided t test . Original numbers used to generate the histogram and number of counted IdU signals (in parentheses) are shown in the right.

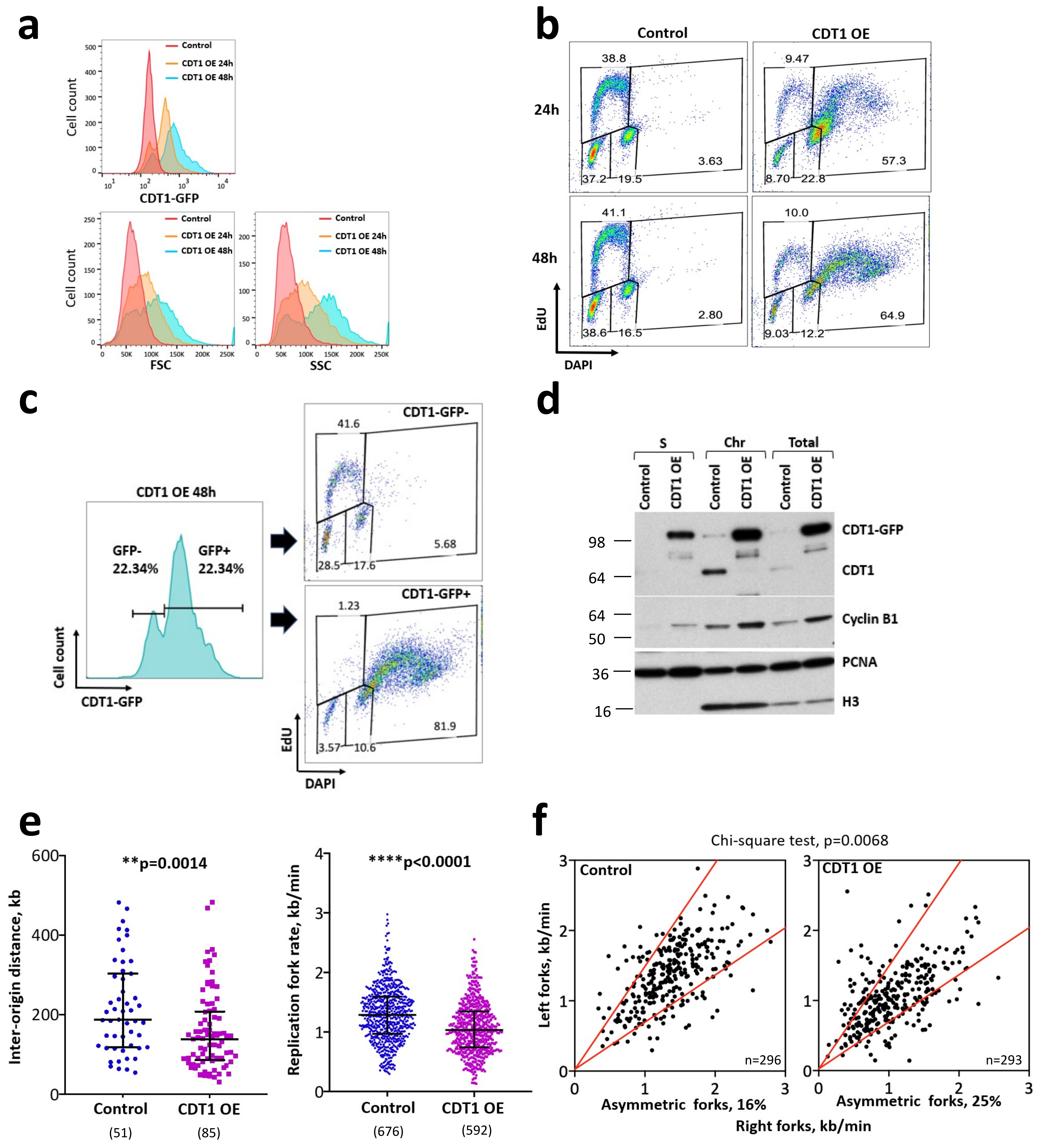
a
Cell count
Control
CDT1 OE 24h
CDT1 OE 48h
CDT1-GFP
FSC
SSC
b
Control
CDT1 OE
24h
48h
38.8
37.2
19.5
3.63
9.47
8.70
22.8
57.3
41.1
38.6
16.5
2.80
10.0
9.03
12.2
64.9
EdU
DAPI
c
CDT1 OE 48h
GFP-
22.34%
GFP+
22.34%
CDT1-GFP
CDT1-GFP-
41.6
28.5
17.6
5.68
CDT1-GFP+
1.23
3.57
10.6
81.9
d
S
Chr
Total
Control
CDT1 OE
98
64
50
36
16
CDT1-GFP
CDT1
Cyclin B1
PCNA
H3
e
Inter-origin distance, kb
**p=0.0014
Control
CDT1 OE
(51)
(85)
Replication fork rate, kb/min
****p<0.0001
(676)
(592)
f
Chi-square test, p=0.0068
Control
CDT1 OE
Left forks, kb/min
n=296
n=293
Asymmetric forks, 16%
Asymmetric forks, 25%
Right forks, kb/min

Supplementary Fig. 3. CDT1 overexpression induces re-replication accompanied by altered replication fork dynamics. A doxycycline inducible CDT1-GFP plasmid was stably transfected into U2OS cells (inducible CDT1-U2OS cells). **a** Inducible CDT1-U2OS cells were cultured without (control) or with (CDT1 OE) doxycycline (1ug/ml) for 24h and 48h. CDT1-GFP expression levels (top), cell size (forward scatter: FSC, bottom left panel) and cell granularity (side scatter: SSC, bottom right panel) were measured by flow cytometry. **b** Cell cycle progression of inducible CDT1-U2OS cells treated with doxycycline for 24h (top) and 48h (bottom). **c** Inducible CDT1-U2OS cells treated with doxycycline for 48h (**b** bottom) were gated based on CDT1-GFP levels (left). Cell cycle analyses of CDT1-GFP negative and positive cells (right) confirmed that cells that lost CDT1-GFP expression did not undergo re-replication. **d** CyclinB1 levels in CDT1 OE cells treated with doxycycline for 48h. Western blot to detect changes of CyclinB1 levels in inducible CDT1-U2OS cell treated with doxycycline for 48 hours. Chr: chromatin enrichment fraction after pre-extraction with 0.25% NP40 of low salt solution; Total: whole cells lysate. **e**, **f** DNA replication profiles of inducible CDT1-U2OS cells with and without doxycycline for 48h were determined using DNA combing. **e** DNA replication fork progression rates (left), inter-replication origin distances (right) are shown with median and interquartile range indicated (total number of fibers counted are shown under the sample name). Mann-Whitney test was used for the statistical significance. $p<0.0001$ for replication fork rate and $p=0.0014$ for origin distance by using a two-sided test, respectively. Similar results were obtained from 2 independent experiments. **f** Asymmetric replication forks in inducible CDT1-U2OS cells treated with doxycycline for 48h . The total number of forks analyzed are shown at the bottom right corner. Percentage of asymmetric forks from each group are shown at the bottom. Chi-square test was used for the statistical significance of the percentage difference of asymmetric fork between control and MLN4924 treated samples.

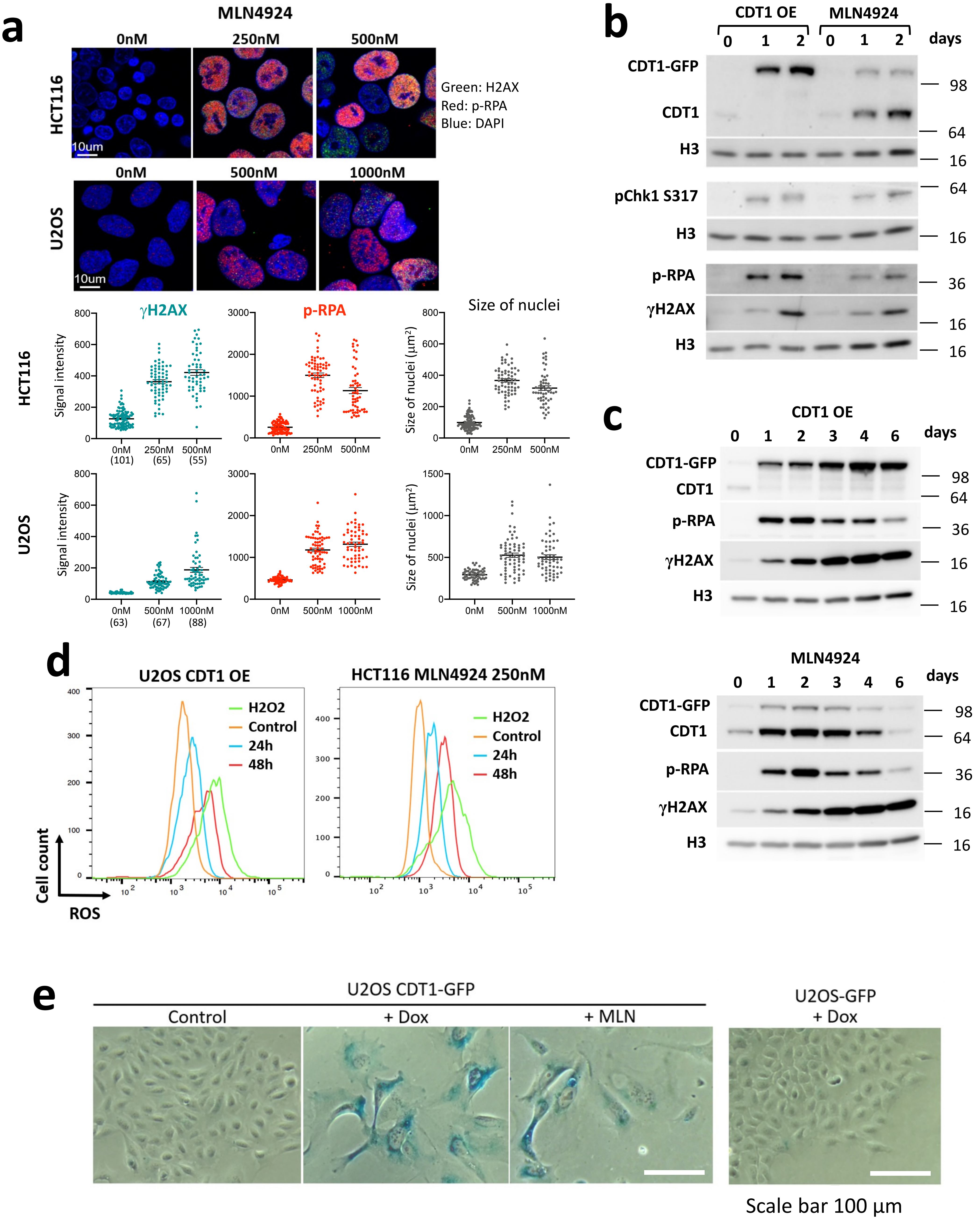
a
MLN4924
0nM
250nM
500nM
HCT116
10um
Green: H2AX
Red: p-RPA
Blue: DAPI
0nM
500nM
1000nM
U2OS
10um
γH2AX
p-RPA
Size of nuclei
HCT116
Signal intensity
Size of nuclei (μm²)
0nM (101)
250nM (65)
500nM (55)
U2OS
0nM (63)
500nM (67)
1000nM (88)
b
CDT1 OE
MLN4924
0 1 2 0 1 2 days
CDT1-GFP
CDT1
H3
pChk1 S317
H3
p-RPA
γH2AX
H3
98
64
16
64
16
36
16
16
c
CDT1 OE
0 1 2 3 4 6 days
CDT1-GFP
CDT1
p-RPA
γH2AX
H3
98
64
36
16
16
MLN4924
0 1 2 3 4 6 days
CDT1-GFP
CDT1
p-RPA
γH2AX
H3
98
64
36
16
16
d
U2OS CDT1 OE
HCT116 MLN4924 250nM
H2O2
Control
24h
48h
Cell count
ROS
e
U2OS CDT1-GFP
Control
+ Dox
+ MLN
U2OS-GFP
+ Dox
Scale bar 100 µm

Supplementary Fig. 4. **DNA re-replication leads to replication stress, activated checkpoint and senescence. a** *Top panel,* representative images of HCT116 and U2OS cells treated with the indicated doses of MLN4924 for 48h. Cells were stained with p-RPA (red) or γH2AX (green). DNA was counterstained with DAPI. *Bottom panel*, Fluorescence intensities (arbitrary units) for p-RPA or γH2AX and nuclei size (μm^2) are shown as dot plot with mean +/- SEM indicated. 2 biological repeats got similar results**.** The total number of cells analyzed (same for γH2AX, p-RPA and size of nuclei) are shown under the γH2AX panels for both HCT116 and U2OS cells**. b** Inducible CDT1-U2OS cells were treated with doxycycline or 500nM of MLN4924 for 1 day and 2 days and changes in p-RPA, γH2AX and pChk1 S317 levels were monitored by immunoblotting. Histone H3 was used as loading control. 2 biological repeats got similar results. **c** Inducible CDT1-U2OS cells were treated with doxycycline for the indicated times and changes in levels of p-RPA, γH2AX and CDT1 were monitored by immunoblotting. Histone H3 was used as loading control. 2 biological repeats got similar results **. d** Inducible CDT1-U2OS cells were treated with 1μg/ml doxycycline (U2OS CDT1 OE, left) and HCT116 cells were treated with 250nM MLN4924 (HCT116, right) for the indicated times and then incubated with the ROS Deep Red Dye probe for 1 h prior flow analysis. Flow cytometry was performed immediately after harvesting cells to measure ROS levels. As a positive control, cells were treated with 1mM H2O2 together with the ROS Deep Red Dye probe. **e** Inducible CDT1-GFP U2OS cells were treated with 1μg/ml doxycycline or 500nM of MLN4924 for 6 days, then stained with β-galactosidase to detect senescence. Inducible GFP U2OS cells were treated with 1μg/ml doxycycline for 6 days as control. 2 biological repeats got similar results.

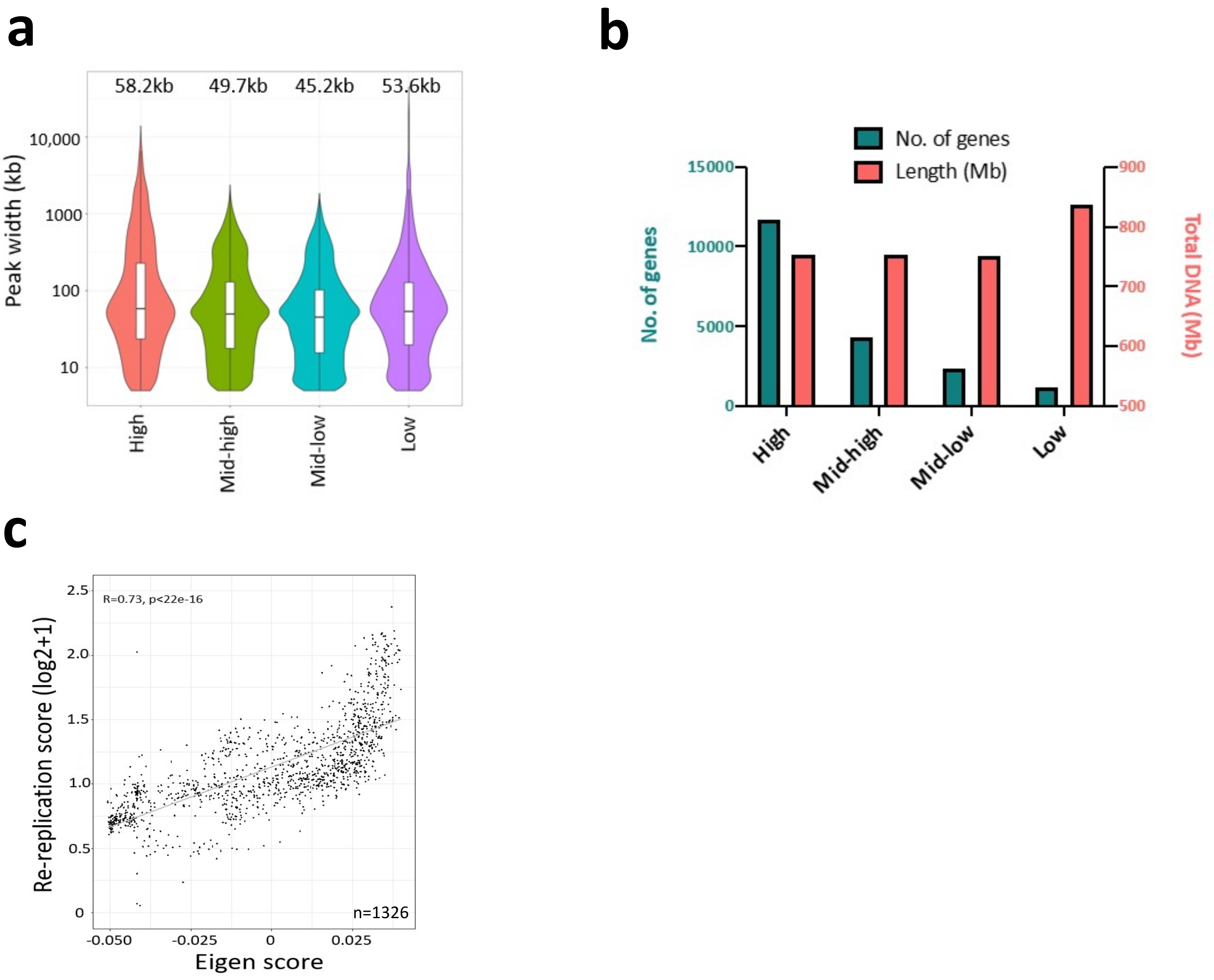

Supplementary Fig. 5. Fragment size, gene density and chromatin condensation of re-replicated DNA. a Size distribution of peak width of the four groups stratified by peak height shown in figure 2. The box range is between 25th percentile and 75th percentile. The whisker length is defined as 1.5 * IQR (Interquartile range). Similar results were obtained from 2 independent experiments. **b** Total DNA length and genes in each of the 4 groups in figure 2. Genes were obtained from TxDB (TxDb.Hsapiens.UCSC.hg19.knownGene) using genes that overlap only one segment type (High, Mid-high, Mid-low, and Low). **c** Dot-plot showed the correlation of binned Eigen scores with re-replicated DNA. Eigen vector bigwig was used as a reference with a 100kb bin sizes. The mean score of the normalized signal of each bin was calculated in the MLN4924 400nM sample. The correlation by Pearson correlation coefficient analysis is 0.73 without outliers. Wilcoxon test was used for the statistical analyses. p<22e-16 by using the default two-sided test.

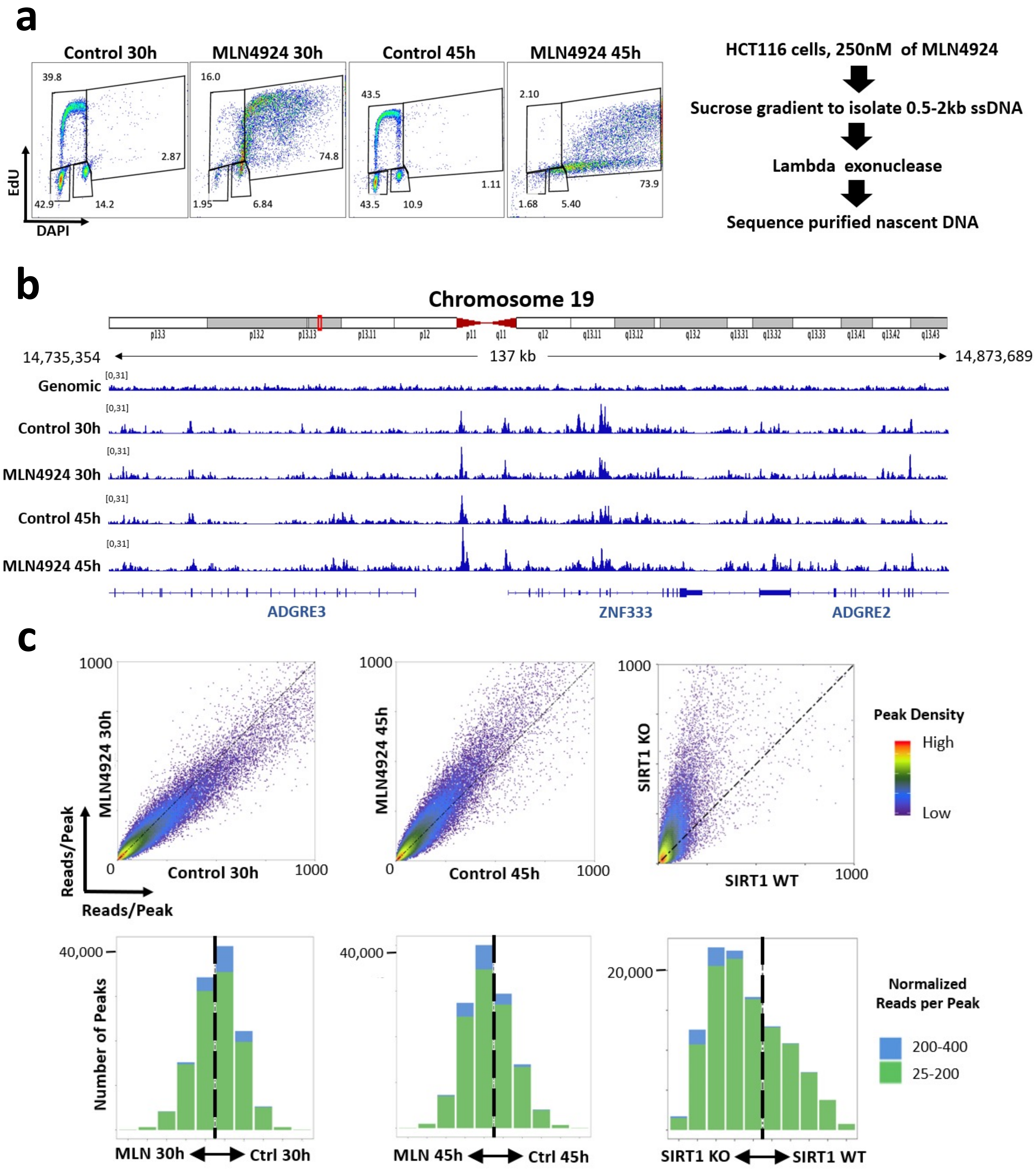
a
Control 30h
MLN4924 30h
Control 45h
MLN4924 45h
39.8
2.87
42.9
14.2
16.0
74.8
1.95
6.84
43.5
1.11
43.5
10.9
2.10
73.9
1.68
5.40
EdU
DAPI
HCT116 cells, 250nM of MLN4924
Sucrose gradient to isolate 0.5-2kb ssDNA
Lambda exonuclease
Sequence purified nascent DNA
b
Chromosome 19
14,735,354
137 kb
14,873,689
Genomic
Control 30h
MLN4924 30h
Control 45h
MLN4924 45h
[0,31]
ADGRE3
ZNF333
ADGRE2
c
MLN4924 30h
Control 30h
MLN4924 45h
Control 45h
SIRT1 KO
SIRT1 WT
1000
0
Reads/Peak
Reads/Peak
Peak Density
High
Low
Number of Peaks
40,000
20,000
MLN 30h
Ctrl 30h
MLN 45h
Ctrl 45h
Normalized
Reads per Peak
200-400
25-200

d

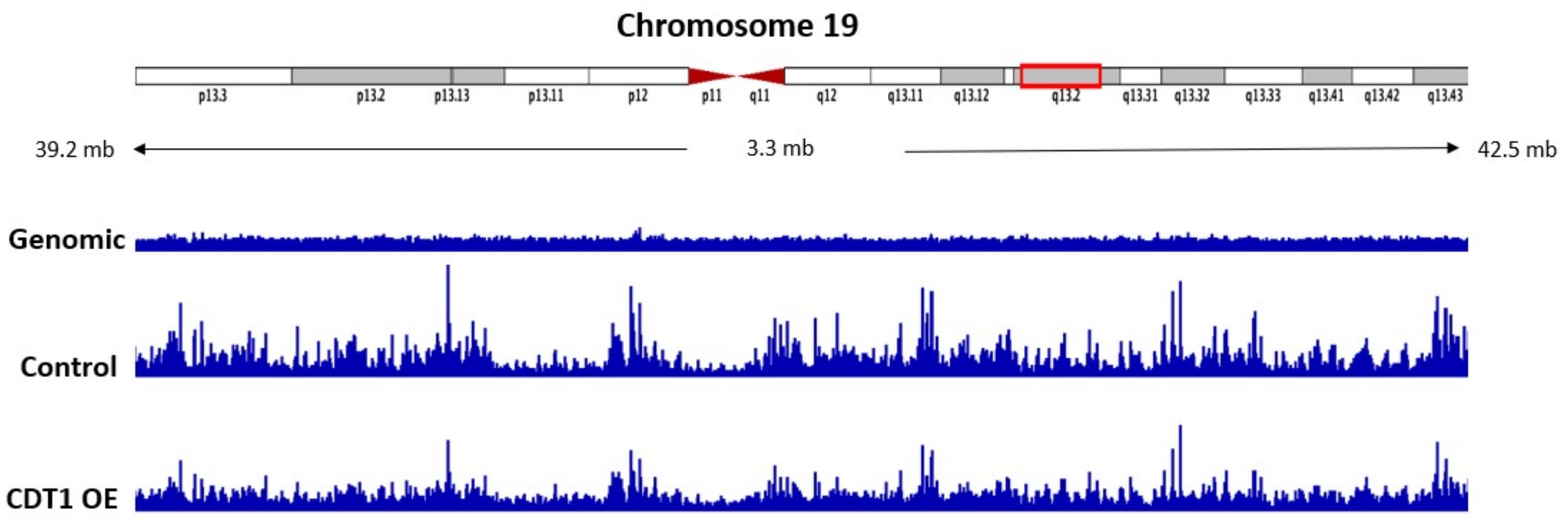

e

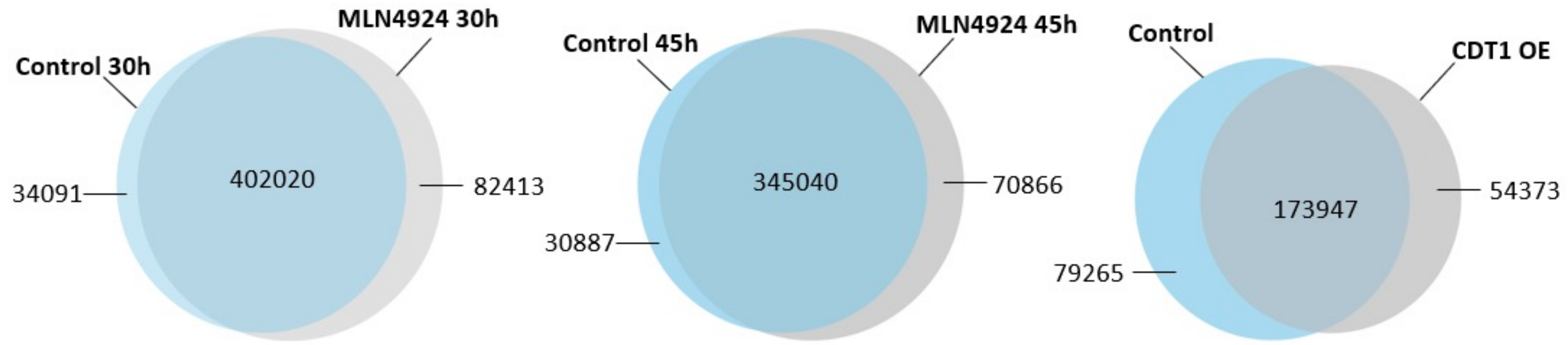

Supplementary Fig. 6. Re-replication is driven by the same set of origins utilized during normal mitotic growth. a To map origins used during re-replication, HCT116 cells were treated with 250nM MLN4924 for 30h and 45h, timepoints for which most of the replicating cells (30h) or nearly all the replicating cells are in a re-replicating cycle (45h). Both control cells and MLN4924-treated cells were collected for nascent strand preparation, followed by next generation whole genome sequencing. Left: 2-dimensional flow cytometry to monitor cell cycle progression; right: experimental flow chart. **b** IGV screenshot of representative nascent-Seq data. Peaks, which represent origins, are similarly distributed in both MLN4924-treated and untreated samples. Genomic: genomic DNA used as background to call replication origin peaks. **c** As in Fig. 4d, density plots (top) and bar graphs (bottom) comparing origin peaks between control cells and MLN4924-treated cells. SIRT1 KO sample was used as a positive control for dormant origin activation. **d, e** U2OS cells harboring doxycycline-inducible CDT1 (CDT1 OE) were treated with doxycycline for 48h for nascent-seq to map origins. **d** IGV screenshot showing origin peaks on Chr19. **e** Venn diagram showing the number of shared peaks between control, MLN4924 (as in c) and CDT1OE samples.

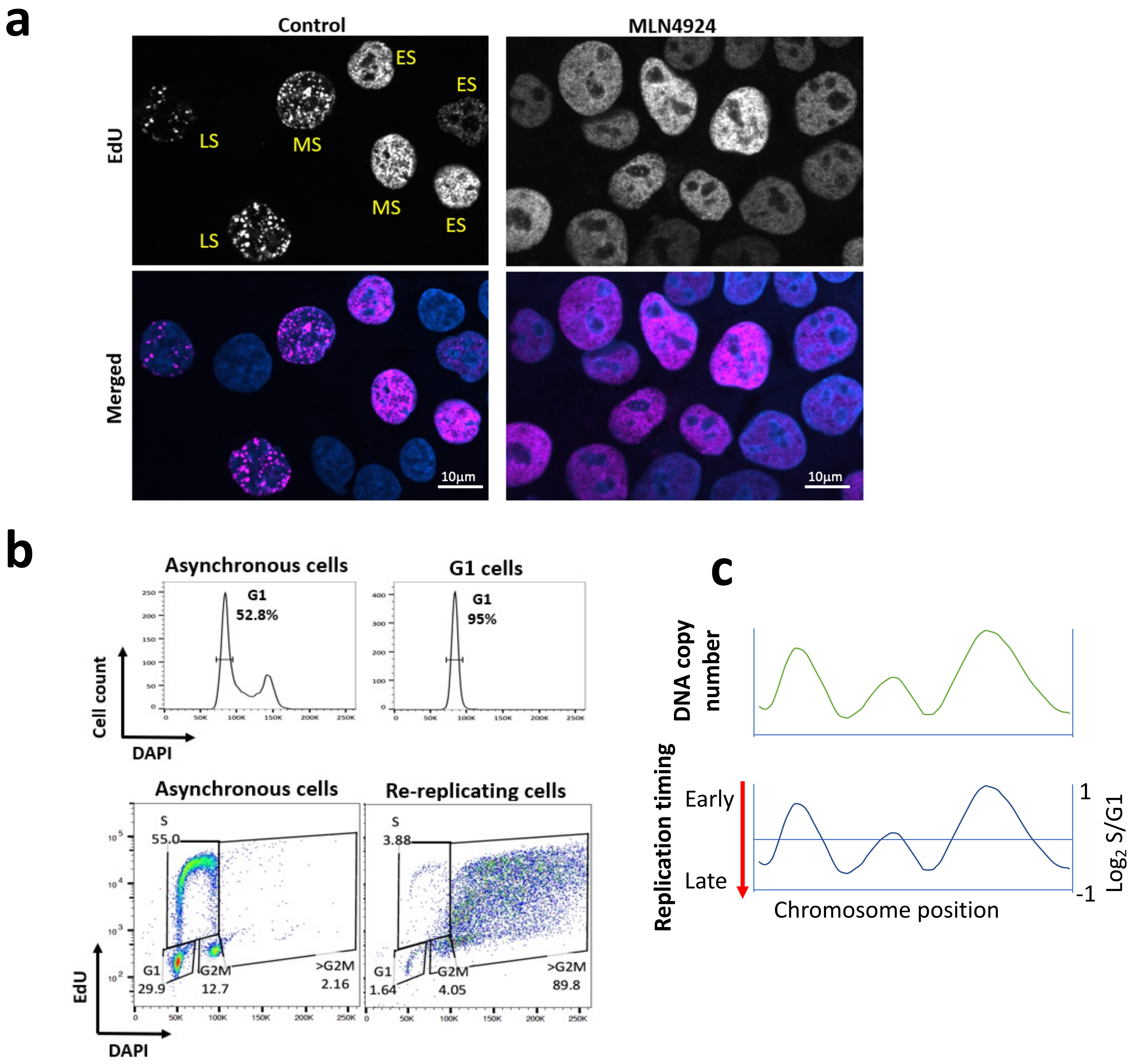

Supplementary Fig. 7. Replication timing distribution of re-replicating DNA. a, HCT116 cells were treated with 250nM of MLN4924 for 30h and EdU click-it to label S phase cells. Control cells showed typical S phase cell foci patterns (ES: early S phase; MS: mid-S phase; LS: late S phase). MLN4924 treated cells lost this typical S phase replication foci patterns, showing ES-like foci patterns. Top, EdU foci; bottom, EdU foci (magenta) merged with DAPI (blue). 3 biological repeats got similar results. **b**, Total genomic DNA of HCT116 cells was purified from G1 cells (isolated by elutriation), from asynchronous normal cell cycle cells and asynchronous re-replicating cells induced by MLN4924 treatment for 36h (when almost all the replicating cells were in a re-replicating cycle) and sequenced at >30X coverage. **c**, Since at any given time in asynchronous cells, early-replicating genomic regions are present in more copies than late-replicating genomic regions (top), the $\log_2$ ratio of copy number in asynchronous cells to that in G1 cells can be used to determine the relative replication timing of any genomic region.

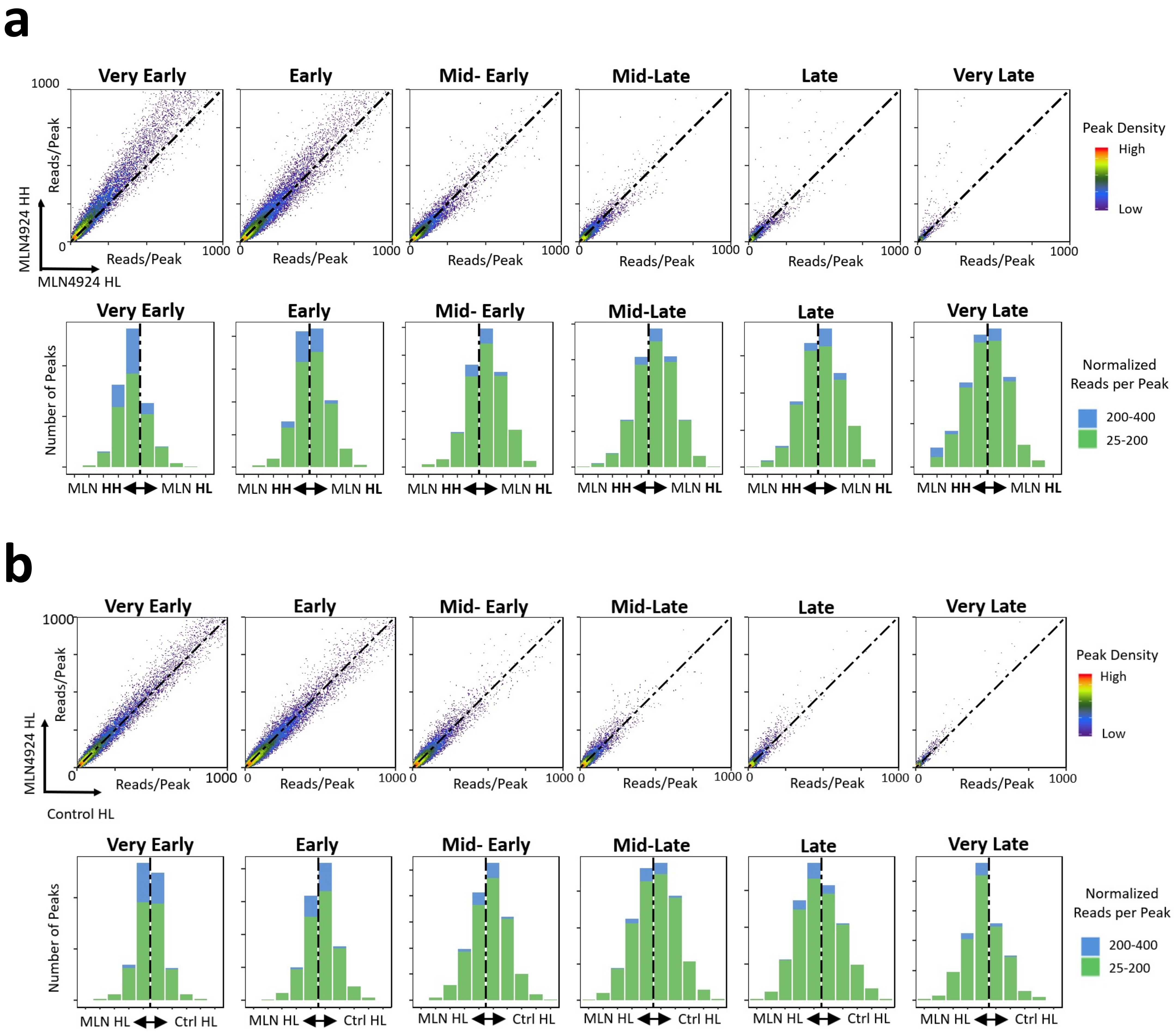

Supplementary Fig. 8. Re-replicating DNA is enriched at early genomic regions. Comparisons of initiation frequencies in re-replicating *vs*. normally replicating origins (MLN-HH versus MLN-HL) (**a**) and in two sets of normally replicating origins (**b**). Density plots and histograms were produced and processed as described in the legend for Fig. 4d.

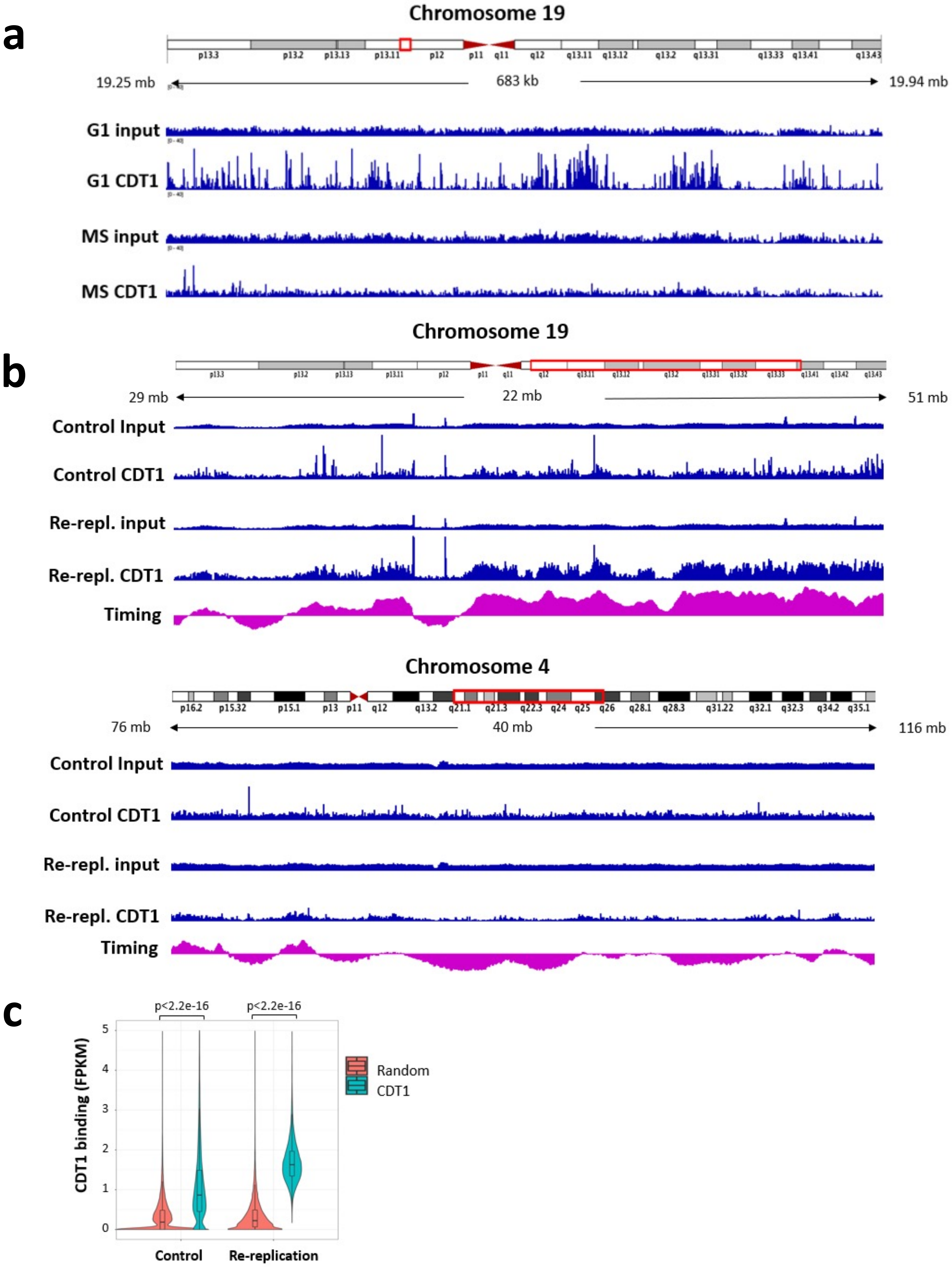
a
Chromosome 19
19.25 mb
683 kb
19.94 mb
G1 input
G1 CDT1
MS input
MS CDT1
b
Chromosome 19
29 mb
22 mb
51 mb
Control Input
Control CDT1
Re-repl. input
Re-repl. CDT1
Timing
Chromosome 4
76 mb
40 mb
116 mb
Control Input
Control CDT1
Re-repl. input
Re-repl. CDT1
Timing
c
p<2.2e-16
p<2.2e-16
CDT1 binding (FPKM)
Random
CDT1
Control
Re-replication

Supplementary Fig. 9. CDT1 is enriched at early origins. a IGV representative screenshot related to the specificity of the CDT1 antibody for ChIP-seq. Double thymidine synchronized HCT116 cells (G1) and mid-S phase HCT116 (MS, released for 6h from double thymidine synchronization) were used for CDT1 ChIP-seq. **b** CDT1 ChIP-seq was performed in HCT116 cells undergoing asynchronous growth (control) and HCT116 cells exposed to MLN4924 for 36 hours (re-replication) as in figure **5a**. IGV screenshots that illustrate the distribution of CDT1 binding sites on parts of chromosome 19 (top) and chromosome 4 (bottom) are shown. Inputs for both control and re-replication and DNA replication timings are shown. **c** CDT1 and randomly shuffled CDT1 binding sites were compared with origin associated CDT1 peaks used in figure **5c** for both control and re-replication. The box range is between 25th percentile and 75th percentile. The whisker length is defined as 1.5 * IQR (Interquartile range). Wilcoxon test was used for the statistical significance analysis. $p<2.2e-16$ by using the default two-sided test for both control and Re-replication samples.

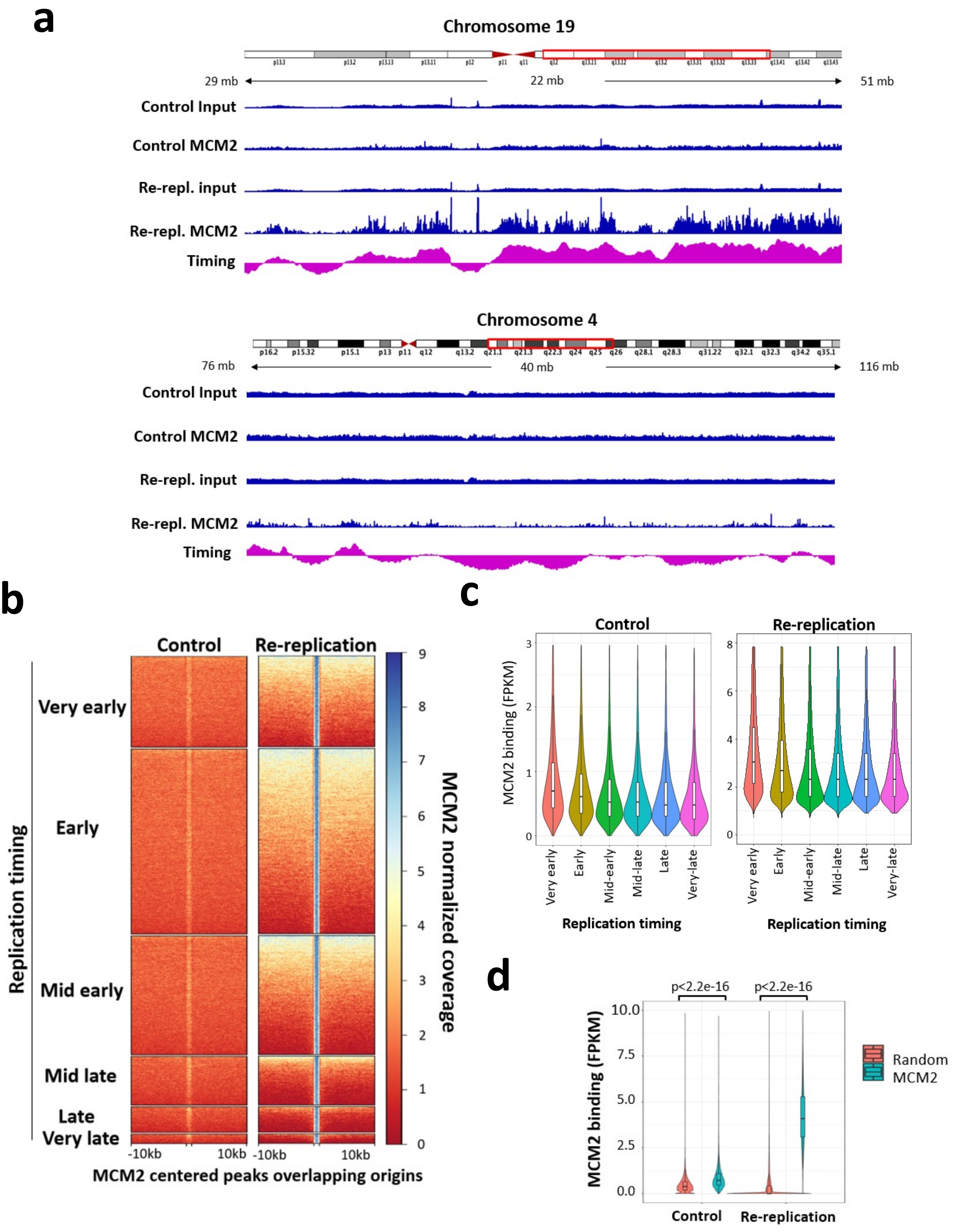

Supplementary Fig. 10 MCM2 is enriched at early origins (Legend on next page).

Supplementary Fig. 10. MCM2 is enriched at early origins. a MCM2-pS139 ChIP-seq was performed in HCT116 cells undergoing asynchronous growth (control) and HCT116 cells exposed to MLN4924 for 36 hours (re-replication). IGV screenshots illustrating the distribution of MCM2-pS139 binding sites on parts of chromosome 19 (top) and chromosome 4 (bottom) in normally proliferating (control) and re-replicating cells are shown. Inputs for both control and re-replication, as well as DNA replication timings of the genomic regions are included. **b** MCM2-pS139 peaks called against input were first intersected with origins (MCM2-pS139 binding sites overlapping with origins), and then divided into 6 groups based on the replication timing data in Fig. 4 to construct a heatmap summarizing the normalized sequencing coverage of MCM2-pS139 ChIP-seq in control and re-replicating samples. **c** Enrichment of MCM2-pS139 centered on replication origins residing in replication timing domains stratified as in **c**, was shown as violin plots. The box range is between 25th percentile and 75th percentile. The whisker length is defined as 1.5 * IQR (Interquartile range). 2 biological repeats got similar results. **d** Randomly shuffled MCM2-pS139 binding sites were compared with origin associated MCM2-p139 peaks used in **c** in both control and re-replication. The box range is between 25th percentile and 75th percentile. The whisker length is defined as 1.5 * IQR (Interquartile range). Wilcoxon test was used for the statistical significance analysis. $p<2.2e-16$ by using the default two-sided test for both control and Re-replication samples. 2 biological repeats got similar results

Supplementary Table 1. Data of Histone modifications from Encode database for Fig. 2 C, D.

Location	Target	Replicate	Sample Type
https://www.encodeproject.org/files/ENCFF522CKP/@@download/ENCFF522CKP.bigWig	H3K27ac	1	BigWig
https://www.encodeproject.org/files/ENCFF445BLD/@@download/ENCFF445BLD.bigWig	H3K27ac	2	BigWig
https://www.encodeproject.org/files/ENCFF413SLE/@@download/ENCFF413SLE.bigWig	H3K27ac	3	BigWig
https://www.encodeproject.org/files/ENCFF547SCJ/@@download/ENCFF547SCJ.bigWig	H3K9ac	1	BigWig
https://www.encodeproject.org/files/ENCFF765VEZ/@@download/ENCFF765VEZ.bigWig	H3K9ac	2	BigWig
https://www.encodeproject.org/files/ENCFF960LSV/@@download/ENCFF960LSV.bigWig	H3K9ac	3	BigWig
https://www.encodeproject.org/files/ENCFF205ZML/@@download/ENCFF205ZML.bigWig	H3K9me3	1	BigWig
https://www.encodeproject.org/files/ENCFF728JJV/@@download/ENCFF728JJV.bigWig	H3K9me3	2	BigWig
https://www.encodeproject.org/files/ENCFF903ELB/@@download/ENCFF903ELB.bigWig	H3K9me3	3	BigWig
https://www.encodeproject.org/files/ENCFF423CUE/@@download/ENCFF423CUE.bigWig	H3K36me3	1	BigWig
https://www.encodeproject.org/files/ENCFF057BUW/@@download/ENCFF057BUW.bigWig	H3K36me3	2	BigWig
https://www.encodeproject.org/files/ENCFF121SMM/@@download/ENCFF121SMM.bigWig	H3K36me3	3	BigWig

Supplementary Table 2. Original data for Fig. 2d

	HH low	HH high	MLN200 low	MLN200 high	MLN400 low	MLN400 high
H3K27ac	0.27	0.36	0.09	0.48	0.08	0.53
H3K9ac	0.27	0.36	0.07	0.52	0.06	0.58
H3K36me3	0.32	0.31	0.05	0.47	0.04	0.61
H3K9me3	0.28	0.37	0.29	0.26	0.32	0.21

Supplementary Table 3. Number of cells at early, mid and late S phase

	Early S	Mid -S	Late S	Total
Control	88 (40.9%)	76 (35.3%)	51 (23.7%)	215
MLN4924	224 (99.6%)	1 (0.4%)	0 (0%)	225

Numbers in brackets are percentage of total.

Supplementary Table 4. CDT1 peak distribution at different replication timing zones for figure 5. % of total peaks in the brackets.

	Peaks per Mb DNA	
Cell cycle	Control (%)	Re-replication (%)
Very early	19.9 (41.2)	201.6 (60.3)
Early	12.4 (25.8)	83.6 (25.0)
Mid-early	4.9 (10.3)	22.9 (6.9)
Mid-late	5.0 (10.4)	15.2 (4.5)
Late	3.8 (7.8)	7.6 (2.3)
Very late	2.2 (4.5)	3.5 (1.0)

Supplementary Table 5. Statistical analysis for Fig. 5c.

Wilcoxon two sample test (A vs B), p value			
A	B	Control	MLN4924
Very early	Very early	1	1
Very early	Early	0.624498742	2.13E-60
Very early	Mid-early	8.98E-163	0
Very early	Mid-late	0	0
Very early	Late	0	0
Very early	Very late	0	0
Early	Early	1	1
Early	Mid-early	1.84E-259	0
Early	Mid-late	0	0
Early	Late	0	0
Early	Very late	0	0
Mid-early	Mid-early	1	1
Mid-early	Mid-late	0	0
Mid-early	Late	0	0
Mid-early	Very late	0	0
Mid-late	Mid-late	1	1
Mid-late	Late	1.53E-121	0
Mid-late	Very late	2.55E-126	0
Late	Late	1	1
Late	Very late	2.10E-13	6.12E-41

Supplementary Table 6. Primer sequence

**Primers**	**Sequence**
**CDT1-F**	5'-TTCCTCCCTTCCTTCTTTCCTTGCTTTC -3'
**CDT1-R**	5'-ATGTCCTACTTGTCCTGCCCAGCTTCTC-3'
**HindIII_Cdt1F**	5'- ATC AAGCTT GGAGCAGCGCCGCGTCAC -3'
**XhoI_Cdt1R**	5'- ATC CTCGAG CAGCCCCTCCTCAGCACGTGTC -3'
**Infusion CDT1F**	5'- CCCTCGTAAAGTCGAATGGATTACAAGGATGACGA -3'
**Infusion CDT1R**	5'- CGATCGATAGATCTGGGCCCCAGCCCCTCCTCAGCAC-3'

Supplementary methods

Western blotting

Most Western blots in this study were done with whole-cell lysates by adding 200μl 1 × SDS loading buffer per 1 million cell pellets. Samples were heated at 100°C for 5 minutes, centrifuged, and the supernatant was used for Western blot. For chromatin bound proteins, cells were incubated on ice for 10 min in a cytosol extraction buffer (10μM HEPES, pH7.9; 10μM KCl; 1μM EGTA; 0.25% NP40; 1X protease inhibitor cocktail and phosphatase inhibitor cocktail), then centrifuged at 2700×*g* for 5 min at 4 °C. The cell pellets were washed once with a cytosol extraction buffer and resuspended in 1XSDS lysis buffer as above. The antibodies used were Phospho-Chk1 (Ser317) (pChk1S317, Cell Signaling, 2344), anti-γH2AX (Millipore, 05-636), anti-p-RPA (Bethyl labs, A300-245A), CDT1 (Cell Signaling, 8064), anti-PCNA (PC10) (Santa Cruz, sc-56) and anti-histone H3 (Millipore, 07-690). ECL signals were detected by x-ray film or ChemiDoc MP Imaging System from BioRad.

β-galactosidase staining

Cells were treated with doxycycline for 7 days, then fixed and stained with a Senescence β-galactosidase Staining Kit (Cell Signaling, 9860) according to the manufacture's instruction. The staining buffer was adjusted to pH6.0 before adding to cells and incubated at 37 °C for overnight.

Reactive Oxygen Species (ROS) in live cells

ROS was detected by Cellular Reactive Oxygen Species Detection Assay Kit (Deep Red Fluorescence, Abcam, ab186029) according to according to the manufacture's manual. In brief, cells were treated with doxycycline or MLN4924 for the indicated times. Cells were incubated with the ROS Deep Red Dye probe for 1 h prior to harvesting. Flow cytometry was performed immediately after harvesting cells to measure ROS levels. As a positive control, 1mM of H2O2 was added to cells together with the ROS Deep Red Dye probe.

Shared peaks and Venn diagram

Shared peaks were identified using a in house BED file intersections script. The script identified peaks which were overlapping within 2kb extension between reference (first file or control) and second file or treated sample. Venn diagram were generated using total number peaks in both files and overlapping peaks as input value for Venn.plot function in Venn Diagram R script.

Re-replication tested by molecular combing

Cells were incubated with CldU for the indicated time and IdU for 30 minutes. Then following the DNA replication analysis by molecular combing method to get combing images. 3-6 images were randomly selected and IdU only and IdU and CldU overlapped (yellow) were counted in Photoshop 2021.
