# Supplementary material for: Dynamics of replication origin over-activation
Source: Nat Commun. 2021 Jun 8;12:3448. doi: 10.1038/s41467-021-23835-0 (PMC8187443; doi:10.1038/s41467-021-23835-0)
Supplement: Supplementary file 3 — Reporting Summary [file 41467_2021_23835_MOESM3_ESM.pdf]

nature research

Corresponding author(s): Mirit I. Aladjem
Last updated by author(s): 4/20/2021

Reporting Summary

Nature Research wishes to improve the reproducibility of the work that we publish. This form provides structure for consistency and transparency in reporting. For further information on Nature Research policies, see our Editorial Policies and the Editorial Policy Checklist.

Statistics

For all statistical analyses, confirm that the following items are present in the figure legend, table legend, main text, or Methods section.

n/a	Confirmed	
☐	☒	The exact sample size (*n*) for each experimental group/condition, given as a discrete number and unit of measurement
☐	☒	A statement on whether measurements were taken from distinct samples or whether the same sample was measured repeatedly
☐	☒	The statistical test(s) used AND whether they are one- or two-sided<br>*Only common tests should be described solely by name; describe more complex techniques in the Methods section.*
☒	☐	A description of all covariates tested
☒	☐	A description of any assumptions or corrections, such as tests of normality and adjustment for multiple comparisons
☐	☒	A full description of the statistical parameters including central tendency (e.g. means) or other basic estimates (e.g. regression coefficient) AND variation (e.g. standard deviation) or associated estimates of uncertainty (e.g. confidence intervals)
☐	☒	For null hypothesis testing, the test statistic (e.g. *F*, *t*, *r*) with confidence intervals, effect sizes, degrees of freedom and *P* value noted<br>*Give P values as exact values whenever suitable.*
☒	☐	For Bayesian analysis, information on the choice of priors and Markov chain Monte Carlo settings
☒	☐	For hierarchical and complex designs, identification of the appropriate level for tests and full reporting of outcomes
☐	☒	Estimates of effect sizes (e.g. Cohen's *d*, Pearson's *r*), indicating how they were calculated

Our web collection on statistics for biologists contains articles on many of the points above.

Software and code

Policy information about availability of computer code

Data collection	1, Flow cytometry data were collected with BD LSR Fortessa cell analyzer with FACSDiva software (version 6.2).<br>2, DNA single fiber images were collected by FiberVision Automated Scanner (Genomic Vision).<br>3, Nascent-seq and replication timing data were collected by using Illumina TruSeq Nano DNA library preparation and paired-end sequencing.<br>4, ChIP-seq data were collected by Illumina NextSeq 75 cycle High Output kit.<br>5, The Zeiss LSM710 confocal microscope and Nikon SoRa super-resolution spinning disk microscope.
Data analysis	1, Flow cytometry data were analyzed by Flowjo 10.6.<br>2, DNA single fiber images were analyzed with FiberStudio (version 0.14, Genomic Vision) and Adobe Photoshop 2021.<br>3, Both nascent seq and CHIP-seq data were analyzed with the following softwares and codes. Trimmomatic (version 0.36) and Trim Galore (version 0.4.5); FastQC (version 0.11.5) [https://www.bioinformatics.babraham.ac.uk/projects/fastqc/], bwa aligner (version 0.7.17);MACS2 (version 2.1.1.20160309); MACS2 metric in R (version 3.5.1); Created R scripts (https://github.com/ncbi/BAMscale/wiki); DRAGEN analysis pipeline (01.003.044.02.05.01.40152); Deeptools(v3.5.0); PlotHeatmap tools (v3.5.0) and Juicer(v1.6).<br>4, Graph Pad Prism 9.0.0. for making graphs and statistical analysis.<br>5, IGV2.80 for sequencing data.<br>6,R ggplot geom_boxplot(3.3.3) for making violin plots for sequencing data.

For manuscripts utilizing custom algorithms or software that are central to the research but not yet described in published literature, software must be made available to editors and reviewers. We strongly encourage code deposition in a community repository (e.g. GitHub). See the Nature Research guidelines for submitting code & software for further information.

Data

Policy information about availability of data

All manuscripts must include a data availability statement. This statement should provide the following information, where applicable:

- Accession codes, unique identifiers, or web links for publicly available datasets
- A list of figures that have associated raw data
- A description of any restrictions on data availability

All the sequencing data were deposited in GEO (GSE172417) . Source data are provided with this paper: Histone modification ChIP-seq data from ENCODE database and Hi-C data from GEO (GSM2795535). The source data underlying Figs. 1g, 1h; 2e; 4c and 5c and Supplementary Figs. 3e, 3f; 4a; 5a, 5c; 9c and 10c, 10d are provided as Source Data files. All data within the manuscript are available from the authors upon request.

Field-specific reporting

Please select the one below that is the best fit for your research. If you are not sure, read the appropriate sections before making your selection.

☒ Life sciences ☐ Behavioural & social sciences ☐ Ecological, evolutionary & environmental sciences

For a reference copy of the document with all sections, see nature.com/documents/nr-reporting-summary-flat.pdf

Life sciences study design

All studies must disclose on these points even when the disclosure is negative.

Sample size	For DNA combing analysis and microscopy cell counting, we analyze 50 to 300 signals/cells that gave sufficient statistics for the effect sizes of interest.
Data exclusions	No data were excluded from analysis.
Replication	For all the experiments, we did at least three independent biological replicates except for sequencing experiments, which have at least 2 independent biological replicates. Results were consistently replicated across multiple experiments with all replicates generating similar results.
Randomization	For DNA fiber analysis, fibers were random selected by the FiberStudio software. For manually counting of IdU and CldU overlapping signals, 3-6 images were randomly selected. We used cell lines for all the experiments, no human or animal subjects were used in the study. Randomization is not generally used for other experiments.
Blinding	Blinding is also not necessary because the results are quantitative and did not require subjective judgment or interpretation.

Reporting for specific materials, systems and methods

We require information from authors about some types of materials, experimental systems and methods used in many studies. Here, indicate whether each material, system or method listed is relevant to your study. If you are not sure if a list item applies to your research, read the appropriate section before selecting a response.

Materials & experimental systems

n/a	Involved in the study	
☐	☒	Antibodies
☐	☒	Eukaryotic cell lines
☒	☐	Palaeontology and archaeology
☒	☐	Animals and other organisms
☒	☐	Human research participants
☒	☐	Clinical data
☒	☐	Dual use research of concern

Methods

n/a	Involved in the study	
☐	☒	ChIP-seq
☐	☒	Flow cytometry
☒	☐	MRI-based neuroimaging

Antibodies

Antibodies used	Primary antibodies:<br>gammaH2AX (Millipore, 05-636), Phospho-RPA (Bethyl labs, A300-245A), Phospho-MCM2 (Ser139) (Cell Signaling, 12958), CDT1 (Cell Signaling, 8064), BrdU (IgG1, Becton Dickinson, 347580), BrdU (Accurate chemical, OBT0030), single-stranded DNA (ssDNA) (IgG 2a, Millipore, MAB3034), histone H3 (Millipore, 07-690) and PCNA (PC10) (Santa Cruz, sc-56) and Phospho-Chk1 (Ser317) (Cell Signaling, 2344)

	Secondary antibodies:<br>Alexa 488 conjugated anti-mouse IgG, Alexa 488 conjugated anti-rabbit IgG and Alexa 568 conjugated anti-rabbit IgG (Thermo Fisher Scientific, A11029, A11008 and A21428), Cy3 (Abcam ab6946), goat anti-rat Cy5 (Abcam, ab6565) and goat anti-mouse BV480 (Jackson ImmunoResearch, 115-685-166).
Validation	All antibodies except antibodies for DNA combing were validated using immunoblots based on the molecular weight of the target. GammaH2AX, Phospho-RPA, PCNA Histone H3 and Phospho-Chk1 antibodies also have been used by many people with many publications. BrdU (IgG1, Becton Dickinson, 347580), BrdU (Accurate chemical, OBT0030) and single-stranded DNA antibodies have been used for combing by many scientists. Phospho-MCM2 (Ser139) was further validated using phosphatase and flow cytometry that bound to the right cell cycle stage (Fig.1e). We validated CDT1 for ChIP-seq by comparing G1 cells and S cells, which have very high CDT1 and very low CDT1 levels, respectively (Supplementary Fig. 9a).

Eukaryotic cell lines

Policy information about cell lines

Cell line source(s)	HCT116(CCL247) and U2OS (HTB96) cell lines, both arefrom ATCC.
Authentication	Doxycycline inducible CDT1 over-expression U2OS cells were validated by western blot and flow cytometry.
Mycoplasma contamination	Mycoplasma tested negative with both cell lines.
Commonly misidentified lines<br>(See ICLAC register)	No commonly misidentified cell lines were used.

ChIP-seq

Data deposition

☒ Confirm that both raw and final processed data have been deposited in a public database such as GEO.

☒ Confirm that you have deposited or provided access to graph files (e.g. BED files) for the called peaks.

Data access links<br>*May remain private before publication.*	*For "Initial submission" or "Revised version" documents, provide reviewer access links. For your "Final submission" document, provide a link to the deposited data.*
Files in database submission	Bam files:<br>HCT116__HH.bam<br>HCT116_14HrLMN200.bam<br>HCT116_14HrLMN400.bam<br>21_genCtrl_S51.sorted.dedup.bam<br>22_genMLN_S52.sorted.dedup.bam<br>17_HLC_S47.sorted.dedup.bam<br>19_HLMLN_S49.sorted.dedup.bam<br>20_HHMLN_S50.sorted.dedup.bam<br>Ctrl-30hrs_S29_QC.bam<br>Ctrl45hrs_QC.bam<br>MLN-30hrs_QC.bam<br>MLN45hrs_QC.bam<br>genHCT116.bam.RG.sorted.markedup.bam<br>2_S2_R1_001.sorted.dedup.bam<br>4_S4_R1_001.sorted.dedup.bam<br>11_S11_R1_001.sorted.dedup.bam<br>12_S12_R1_001.sorted.dedup.bam<br>7_S7_R1_001.sorted.dedup.bam<br>8_S8_R1_001.sorted.dedup.bam<br>MLN1_CDT1.dedup.bam<br>MLN2_CDT1.dedup.bam<br>C1_CDT1.dedup.bam<br>C2_CDT1.dedup.bam<br>MLN1_CDT1.dedup.bam<br>MLN2_CDT1.dedup.bam<br>C1_139.dedup.bam<br>C2_139.dedup.bam<br>M1_139.dedup.bam<br>M2_139.dedup.bam<br>HCT116_G1.bam.RG.sorted.markedup.bam<br>HCT116_S.bam.RG.sorted.markedup.bam<br>HCT116_re_rep.bam.RG.sorted.markedup.bam<br>U1.sorted.dedup<br>U2.sorted.dedup

UD1.sorted.dedup
UD2.sorted.dedup
genU.sorted.dedup

Bed files:
HCT116__HH_segmentation.bed
HCT116_14HrLMN200_segmentation.bed
HCT116_14HrLMN400_segmentation.bed
HLC.bed
HLMLN.bed
HHMLN.bed
Ctrl-30hrs.bed
Ctrl-45hrs.bed
MLN-30hrs.bed
CMLN-45hrs.bed
Ctrl-30hrs_S29_peaks.broadPeak
Ctrl-45_peaks.broadPeak
MLN-30hrs_peaks.broadPeak
MLN45hrs_peaks.broadPeak
C1_CDT1.bed
C2_CDT1.bed
MLNN1_CDT1.bed
MLNN2_CDT1.bed
C1_139.bed
C2_139.bed
M1_139.bed
M2_139.bed
HCT116_S_merged.bam_vs_HCT116_G1_merged.bam.bed
HCT116_S_merged.bam_vs_HCT116_G1_merged.bam.top10bed.bed
HCT116_rerep_merged.bam_vs_HCT116_G1_merged.bam.bed
HCT116_rerep_merged.bam_vs_HCT116_G1_merged.bam.top10bed.bed
U1_peaks.broadPeak
U2_peaks.broadPeak
UD1_peaks.broadPeak
UD2_peaks.broadPeak
genUD.sorted.dedup
2_S2_R1_001.sorted.dedup.bam.scaled.bw BigWig
4_S4_R1_001.sorted.dedup.bam.scaled.bw BigWig
7_S7_R1_001.sorted.dedup.bam.scaled.bw BigWig
8_S8_R1_001.sorted.dedup.bam.scaled.bw BigWig
11_S11_R1_001.sorted.dedup.bam.scaled.bw BigWig
12_S12_R1_001.sorted.dedup.bam.scaled.bw BigWig

Genome browser session
(e.g. UCSC)

No longer applicable

Methodology

Replicates

2 biological replicates

Sequencing depth

Up to 40 million reads from 2 rounds of sequencing with single-end (75bp). More than 95 % of reads were mapped.

Antibodies

Phospho-MCM2 (Ser139) (Cell Signaling, 12958) and CDT1 (Cell Signaling, 8064)

Peak calling parameters

Peaks with high read coverages were identified by the narrow MACS2 (version 2.1.1.20160309) peak calling method using input DNA as controls. Peaks were filtered using the “peak-score” MACS2 metric in R (version 3.5.1) by accepting regions above the inflection point threshold of “peak-scores” from the raw output.

Data quality

Peak calling was performed with MACS, all the peaks were above the FDR threshold of 0.05.

Software

Trimmomatic (version 0.36) and Trim Galore (version 0.4.5); FastQC (version 0.11.5) [https://www.bioinformatics.babraham.ac.uk/projects/fastqc/], bwa aligner (version 0.7.17);MACS2 (version 2.1.1.20160309); MACS2 metric in R (version 3.5.1); Created R scripts (https://github.com/ncbi/BAMscale/wiki).

Flow Cytometry

Plots

Confirm that:

☒ The axis labels state the marker and fluorochrome used (e.g. CD4-FITC).

☒ The axis scales are clearly visible. Include numbers along axes only for bottom left plot of group (a 'group' is an analysis of identical markers).

☒ All plots are contour plots with outliers or pseudocolor plots.

☒ A numerical value for number of cells or percentage (with statistics) is provided.

Methodology

Sample preparation	Cancer cell lines were processed according to the EdU kit. For CDT1 and pMCM2 staining, cells were permeabilized before PFA fixation.
Instrument	BD LSR Fortessa cell analyzer.
Software	FACSDiva software for collecting samples and Flowjo 10.6. for analysis.
Cell population abundance	Since we have background information for the parameter detected, antibodies are very good, there are always both negative and positive populations in the same sample, it's pretty straightforward to gate. Since it's pretty straightforward, to avoid too crowd graph, we did not induce axis scales for all the graphs.
Gating strategy	Single cells gated according to DAPI-H DAPI-A were analyzed as the gates shown on each graphs.

☒ Tick this box to confirm that a figure exemplifying the gating strategy is provided in the Supplementary Information.
