# Supplementary material for: Dynamics of replication origin over-activation
Source: Nat Commun. 2021 Jun 8;12:3448. doi: 10.1038/s41467-021-23835-0 (PMC8187443; doi:10.1038/s41467-021-23835-0)

Whole blots for Supplementary Figure 1d

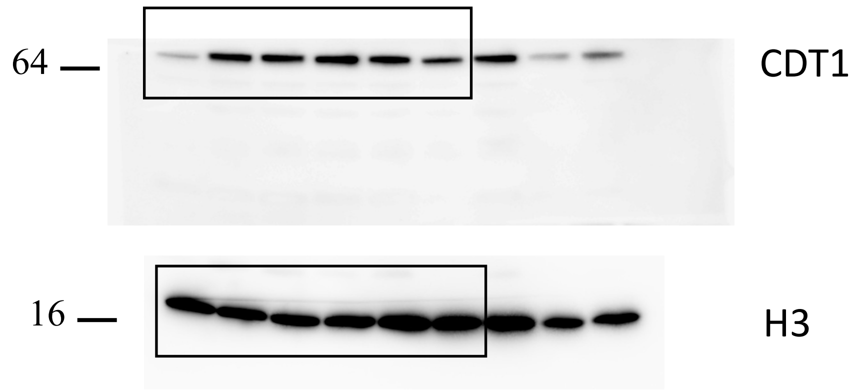

Whole blots for Supplementary Figure 3d

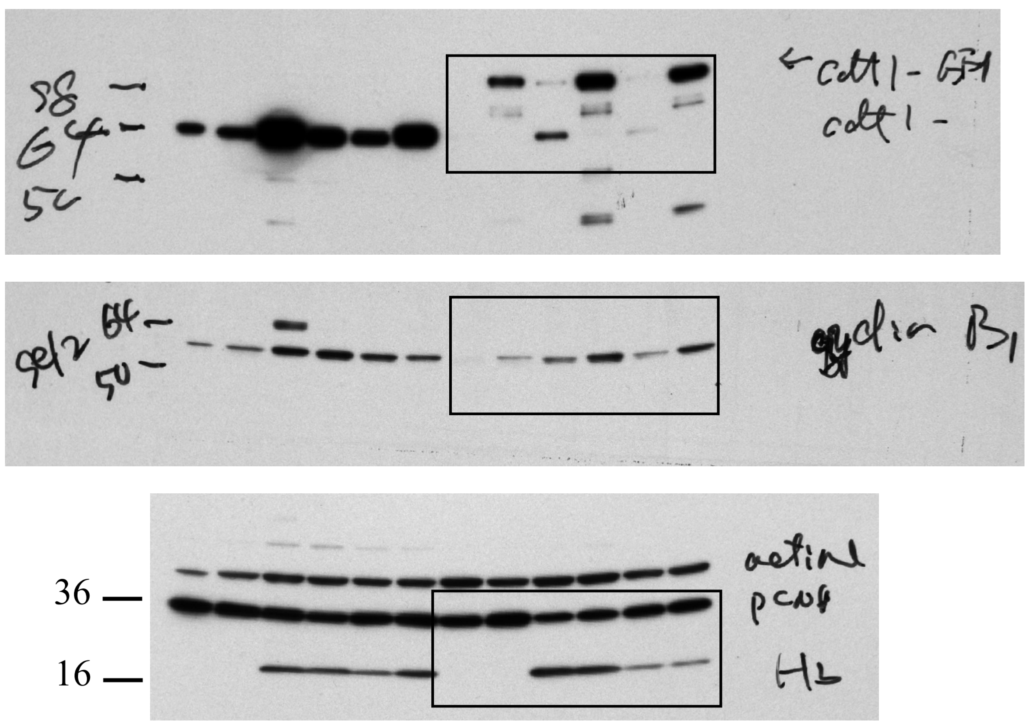

Whole blots for Supplementary Figure 4b

4B

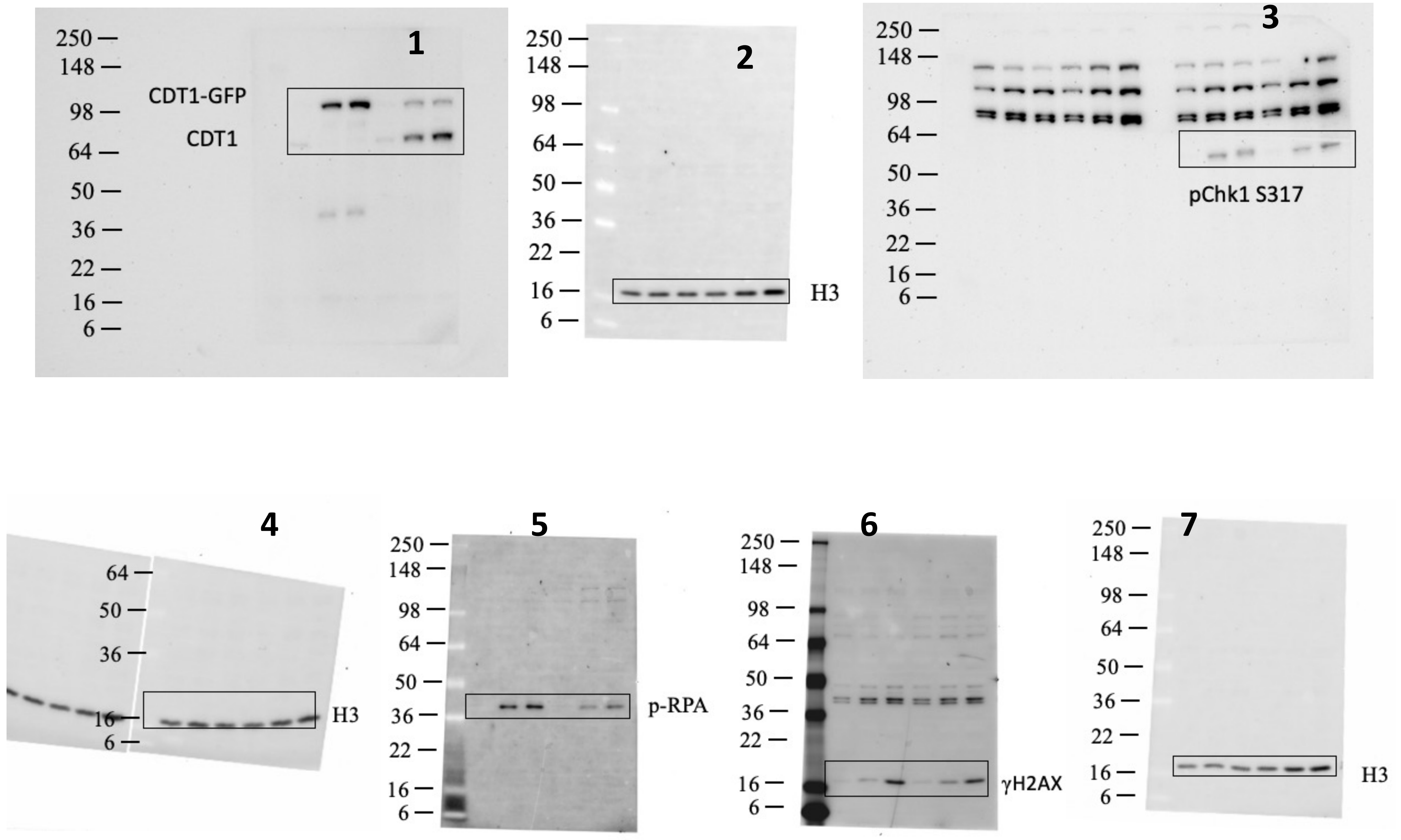

Whole blots for Supplementary Figure 4c

4c top, CDT1 OE

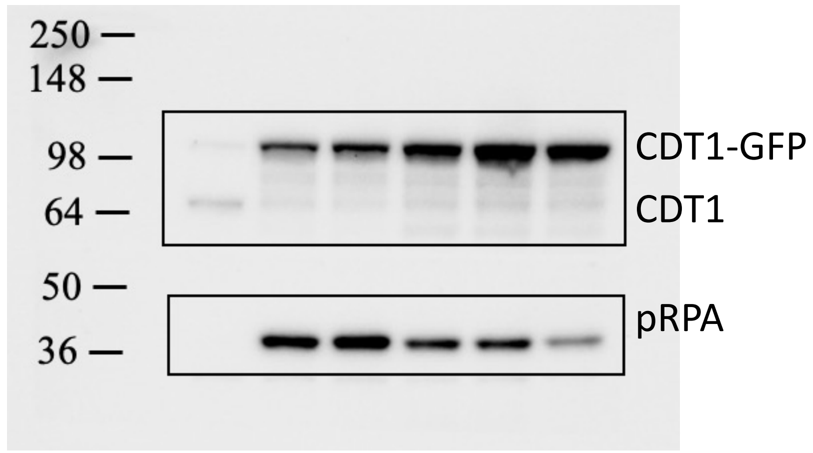

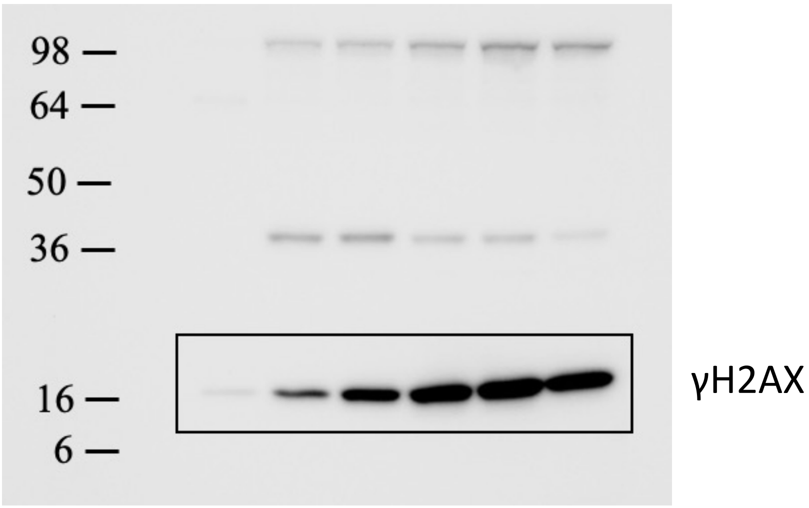

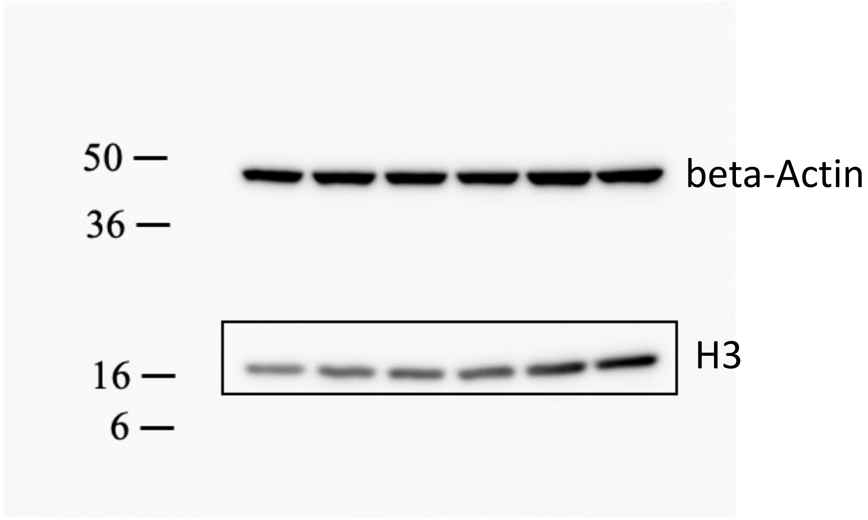

4c bottom, MLN4924

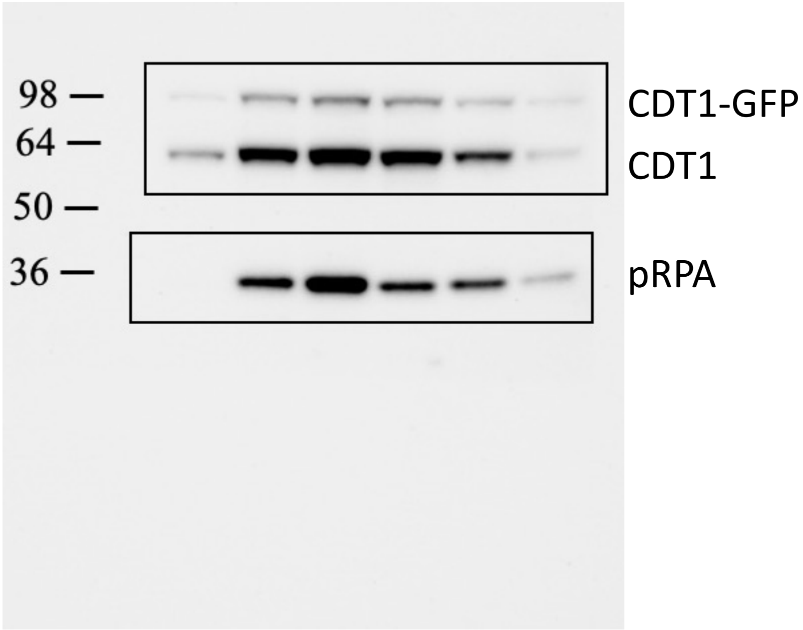

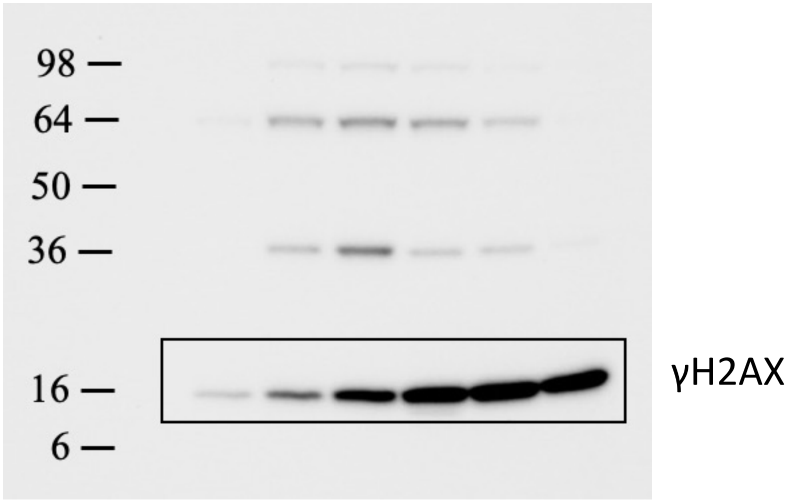

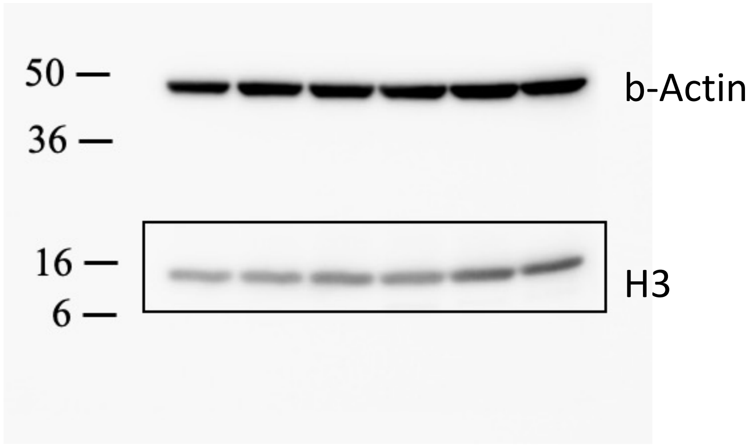

Supplement: Supplementary file 4 — Source Data [file 41467_2021_23835_MOESM4_ESM.zip › source data 051221/Uncroped blot.pdf]
